# Supplementary material for: High-fidelity (repeat) consensus sequences from short reads using combined read clustering and assembly
Source: BMC Genomics. 2024 Jan 24;25:109. doi: 10.1186/s12864-023-09948-4 (PMC10809544; doi:10.1186/s12864-023-09948-4)

**Supplementary dotplots**

30 additional dotplots from 15 repetitive elements (represented by a supercluster) comparing assembled NODEs with RE2 contigs and assembled NODEs with copies on long ONT reads.

For further information see Fig. 2c in the manuscript (SCL008 is included in Fig. 2c and is the first set of dotplots in this supplemental information).

Colors in the following dotplots:

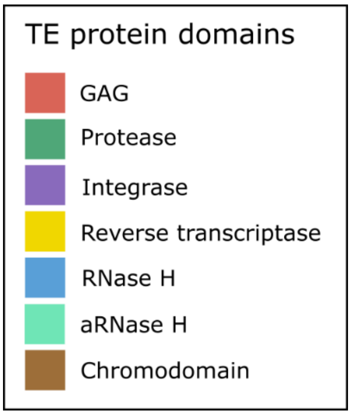


The shading refers to the longest common subsequence (LCS), in which darker grey indicates a longer sequence overlap. In the upper part (above the main diagonal) the forward LCS and in the lower part (below the main diagonal) the reverse LCS is used for shading

Sequence naming scheme in dotplots:

| | |
|---|---|
| NODE … | assembled NODE |
| CL … | RE2 contig |
| [ID] … | ONT read |

| Supercluster | Classification |
| --- | --- |
| SCL008 | LTR/Ty1-copia/Angela |

Assembled NODEs and RE2 contigs

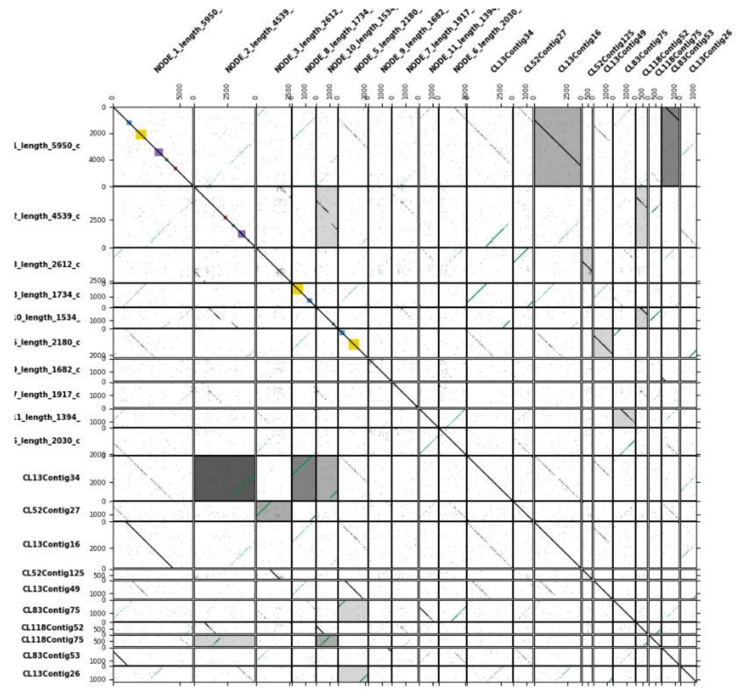

Assembled NODEs and ONT reads (with similar repeat copies)

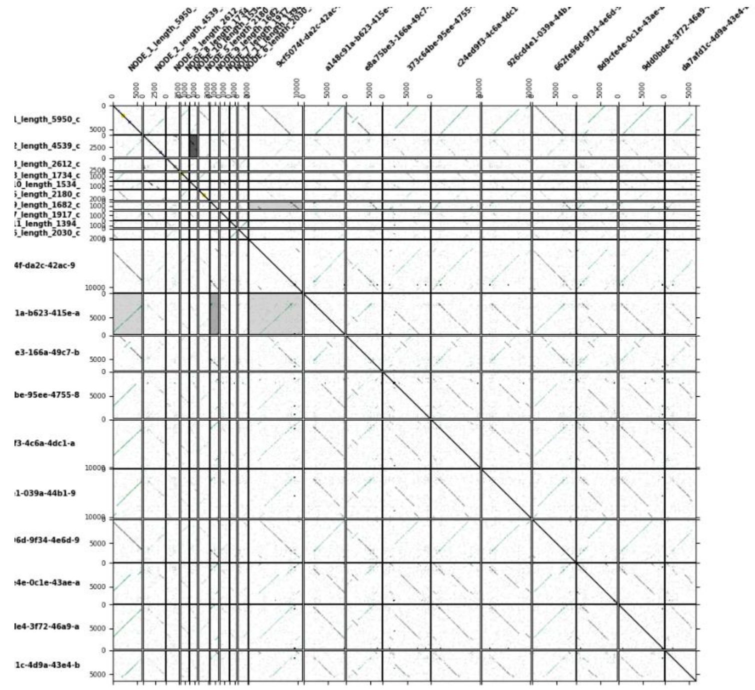

| | |
|---|---|
| Supercluster | Classification |
| SCL001 | LTR/Ty3-gypsy |

Assembled NODEs and RE2 contigs

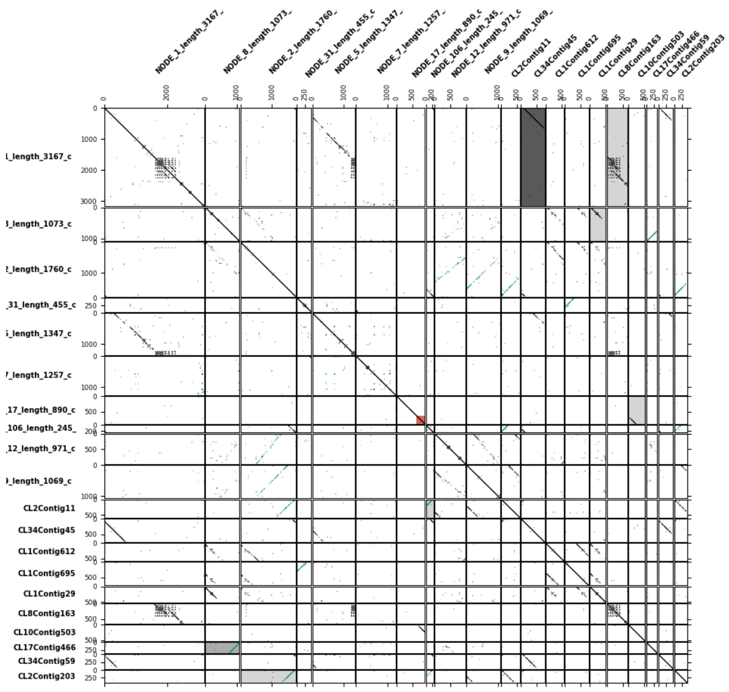


Assembled NODEs and ONT reads (with similar repeat copies)

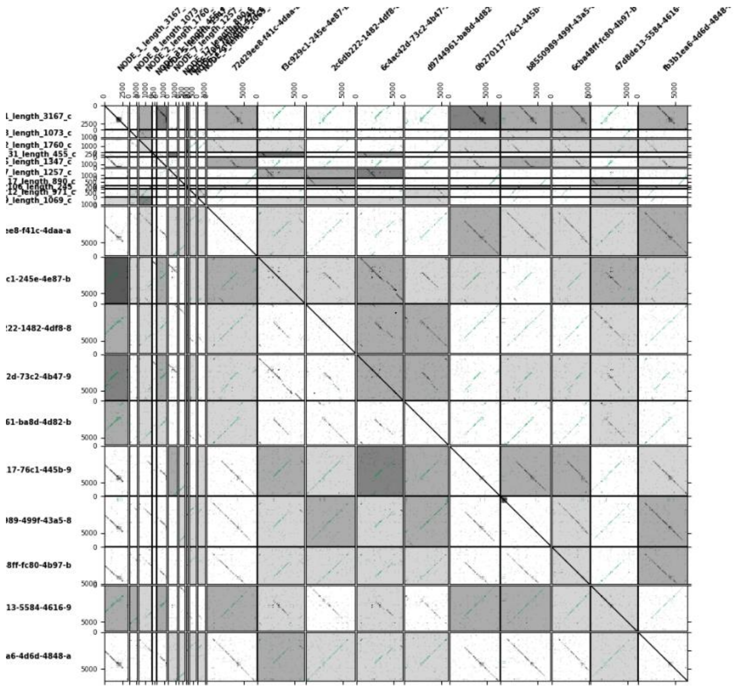

Supercluster | Classification

SCL002 | LTR/Ty1-copia/Athila

Assembled NODEs and RE2 contigs

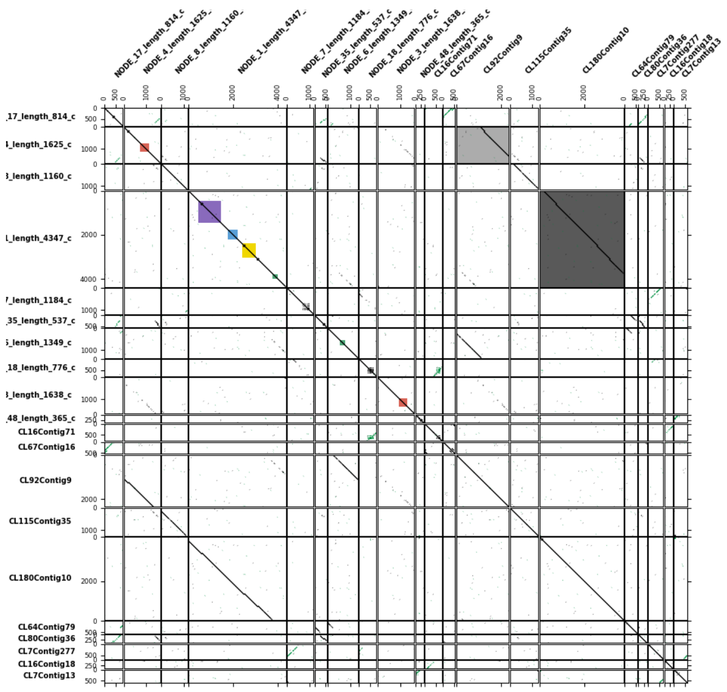

Assembled NODEs and ONT reads (with similar repeat copies)

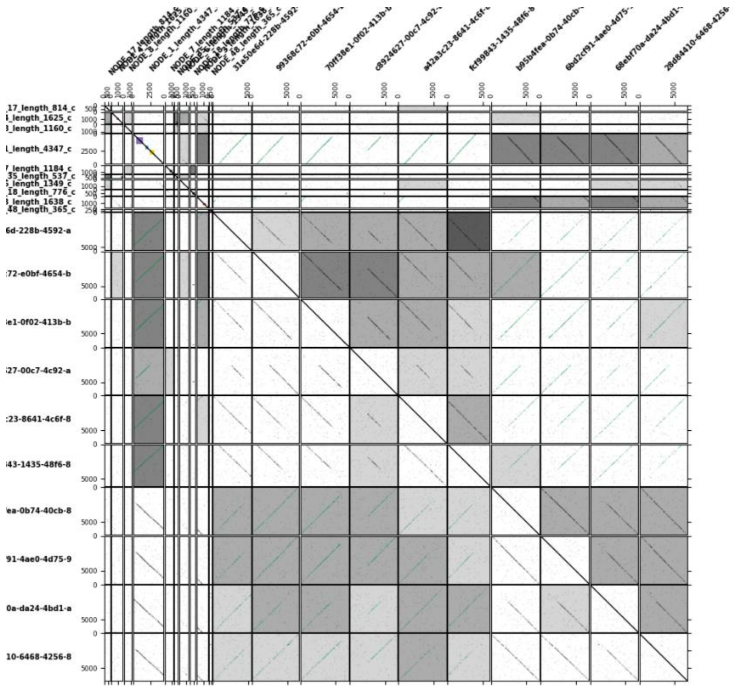

| Supercluster | Classification |
| --- | --- |
| SCL003 | LTR/Ty3-gypsy/Ogre |

Assembled NODEs and RE2 contigs

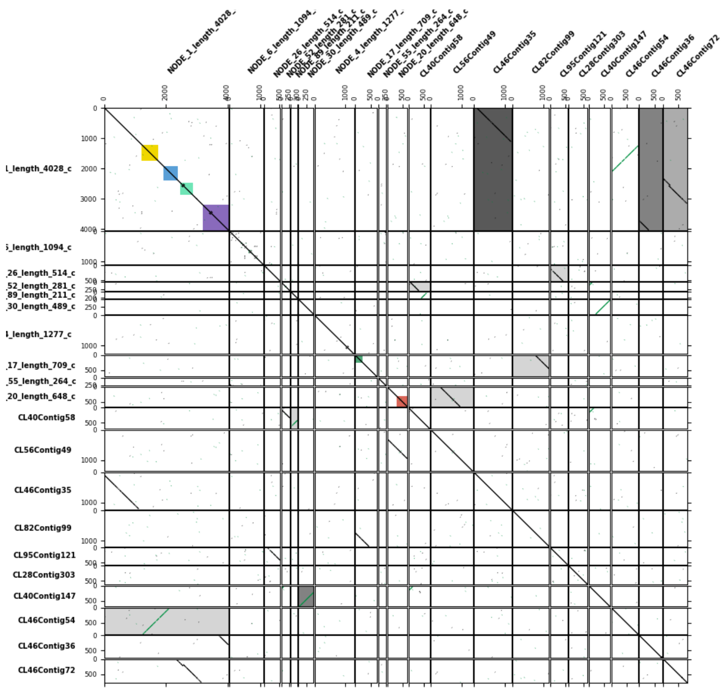


Assembled NODEs and ONT reads (with similar repeat copies)

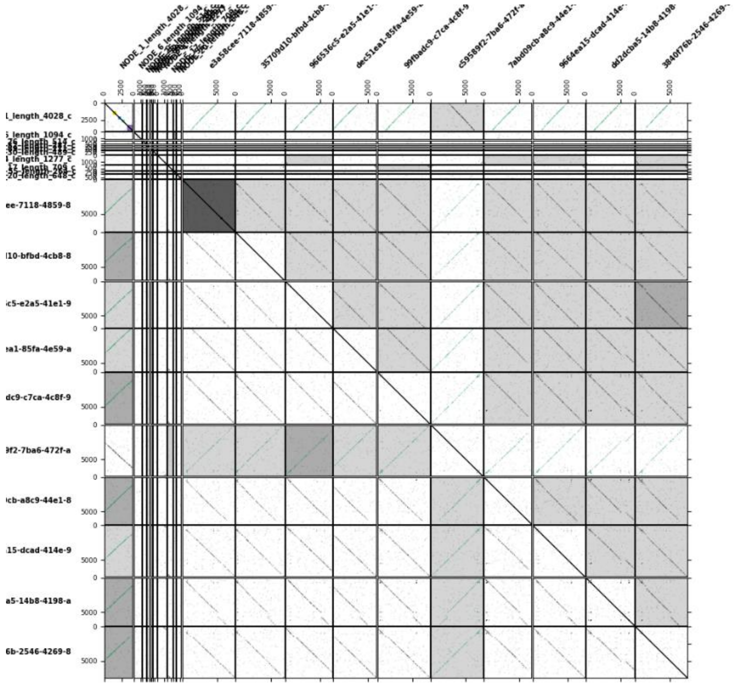

Supercluster | Classification
--- | ---
SCL004 | LTR/Ty3-gypsy/Retand

Assembled NODEs and RE2 contigs

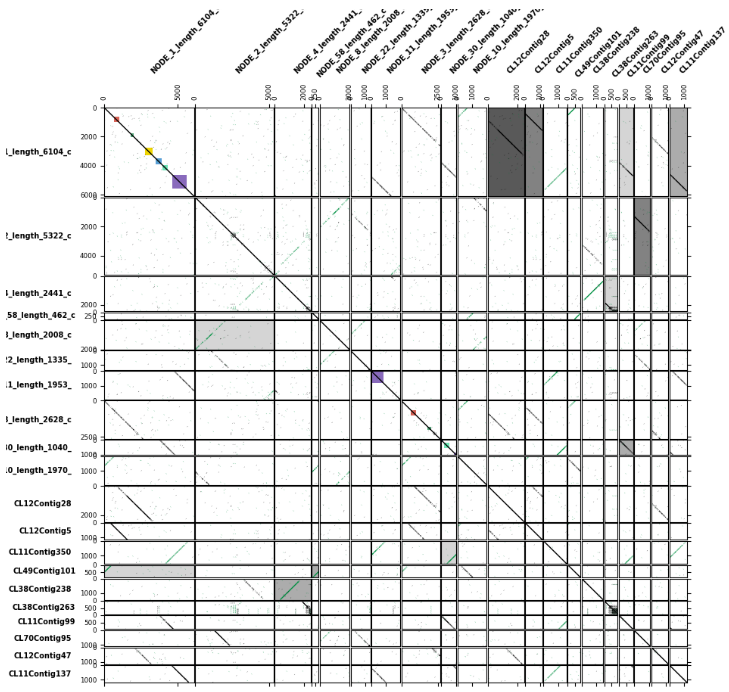


Assembled NODEs and ONT reads (with similar repeat copies)

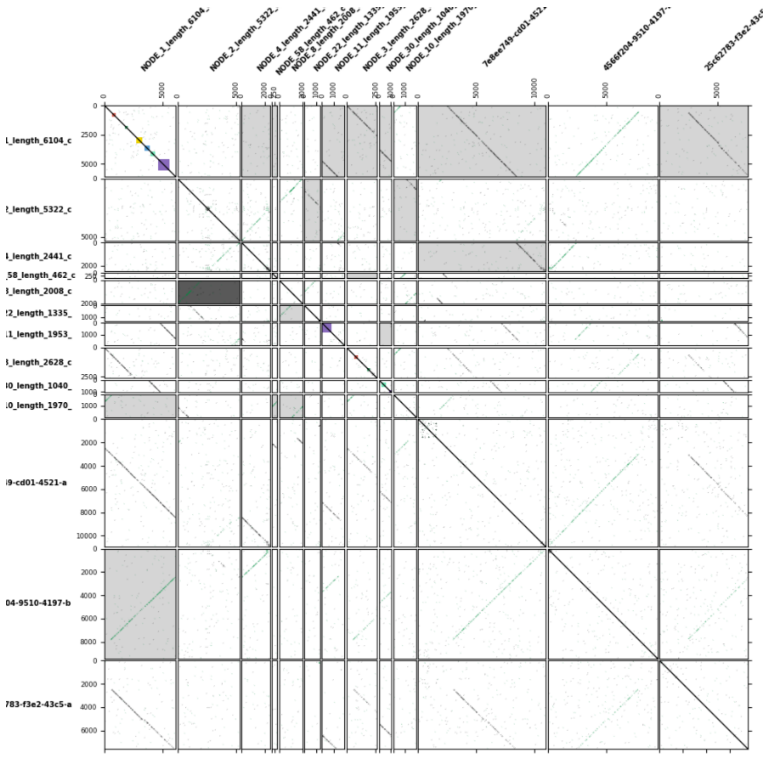

Supercluster | Classification
SCL005 | LTR/Ty3-gypsy/Tekay

Assembled NODEs and RE2 contigs

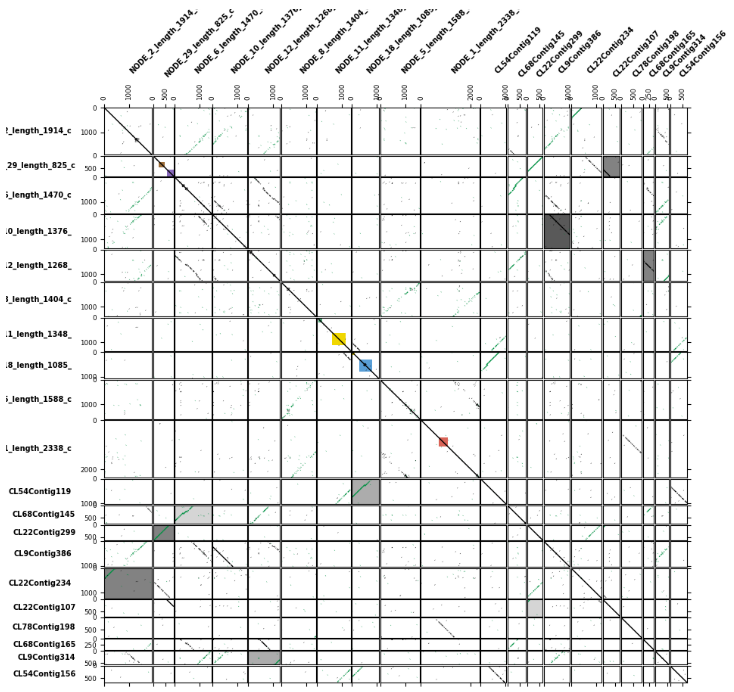


Assembled NODEs and ONT reads (with similar repeat copies)

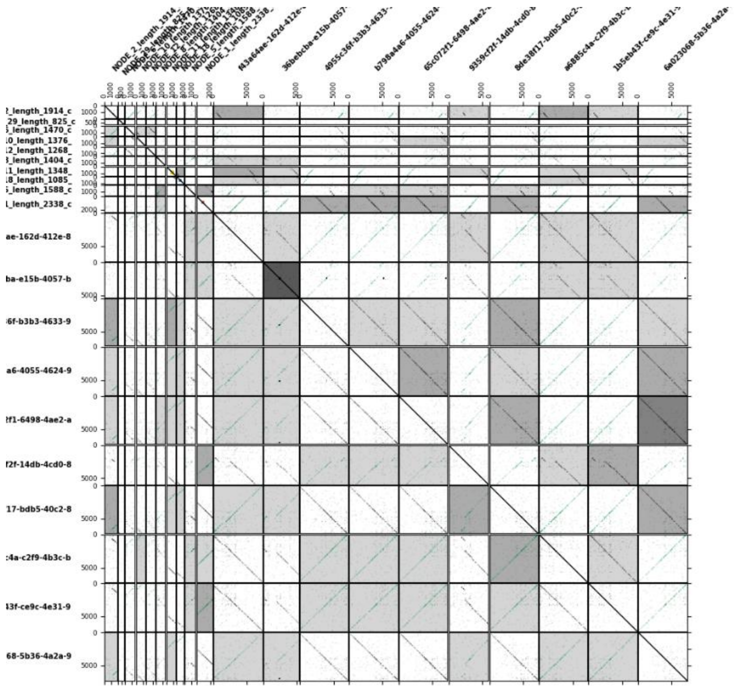

| Supercluster | Classification |
| --- | --- |
| SCL009 | LTR/Ty3-gypsy/Tekay |

Assembled NODEs and RE2 contigs

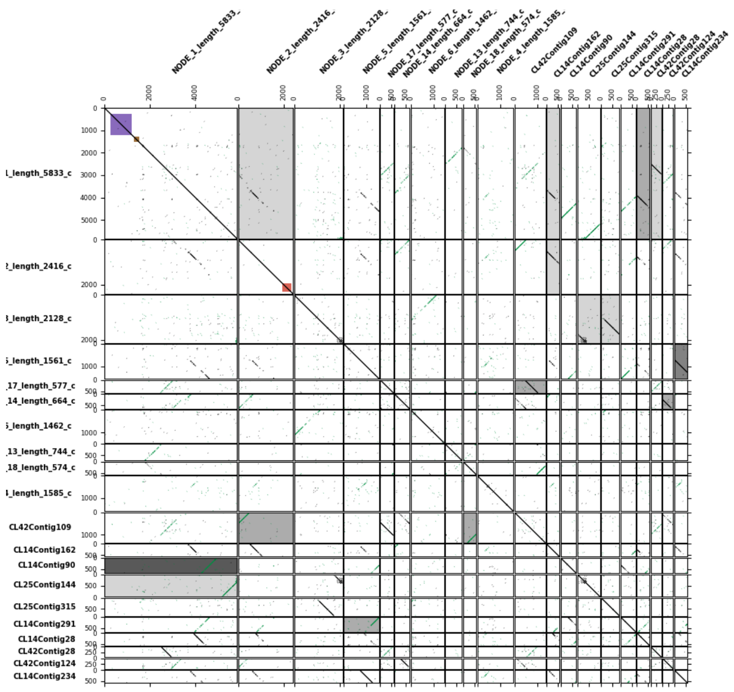

Assembled NODEs and ONT reads (with similar repeat copies)

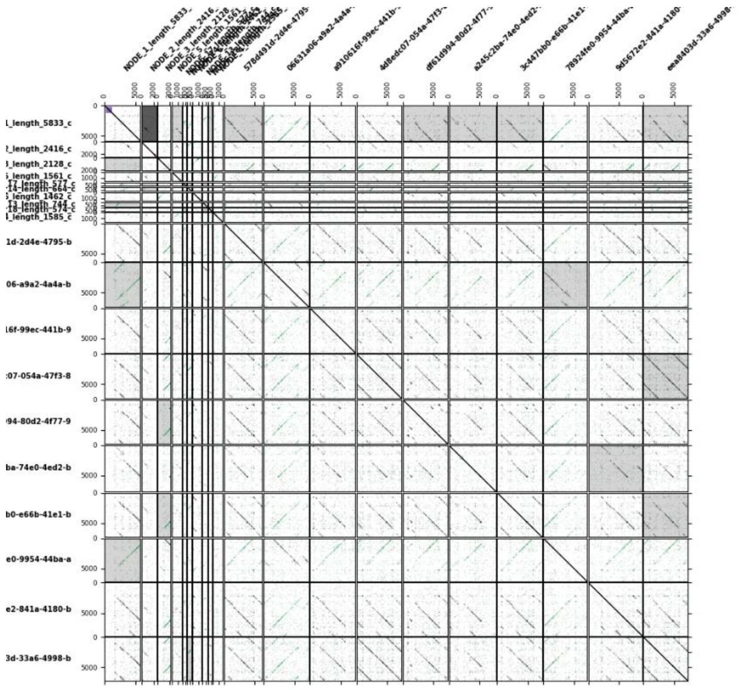

| Supercluster | Classification |
|---|---|
| SCL011 | LTR |

Assembled NODEs and RE2 contigs

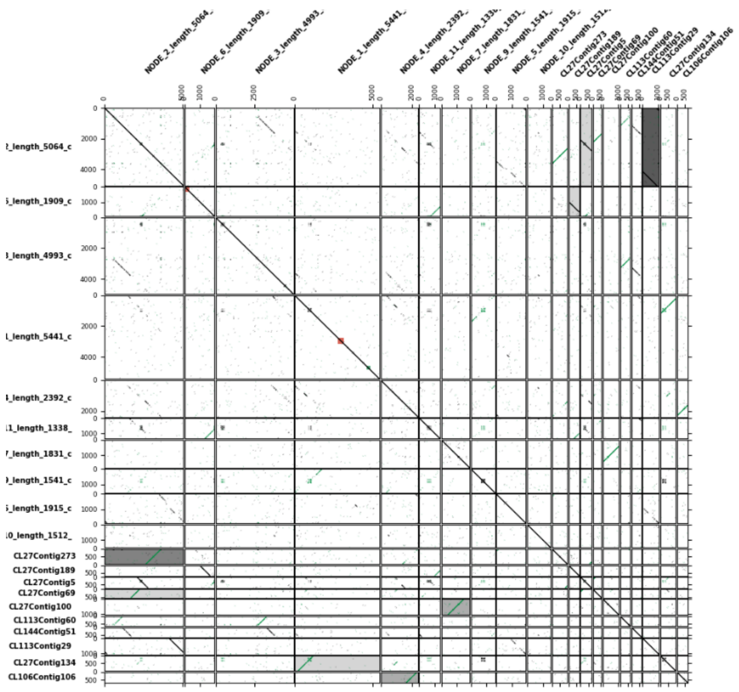


Assembled NODEs and ONT reads (with similar repeat copies)

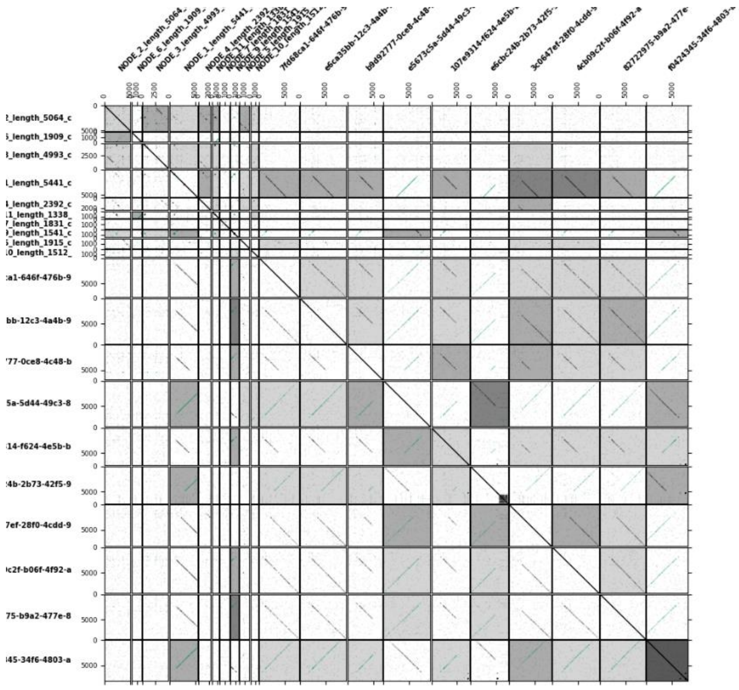

| Supercluster | Classification |
|---|---|
| SCL016 | satellite |

Assembled NODEs and RE2 contigs

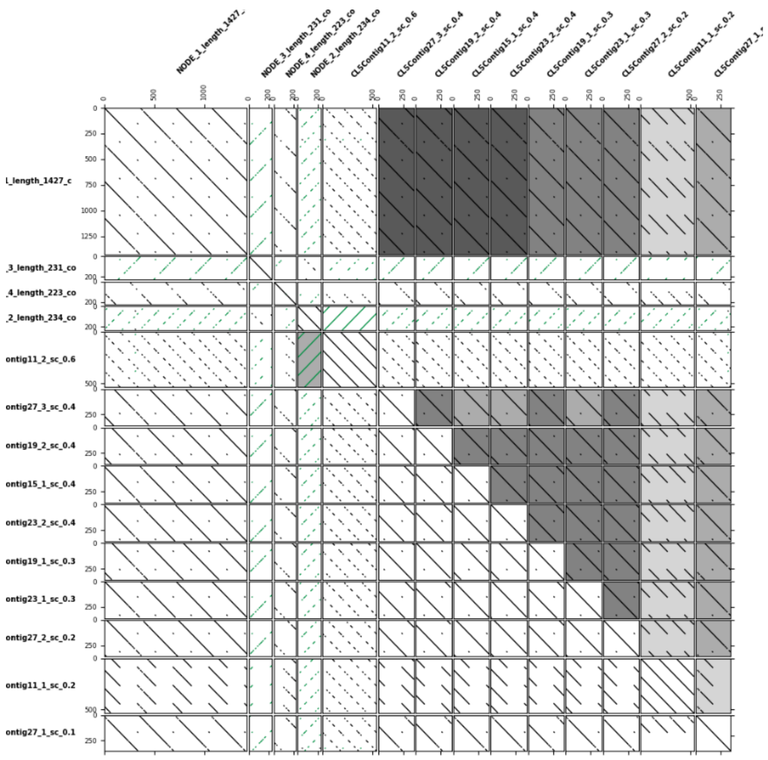


Assembled NODEs and ONT reads (with similar repeat copies)

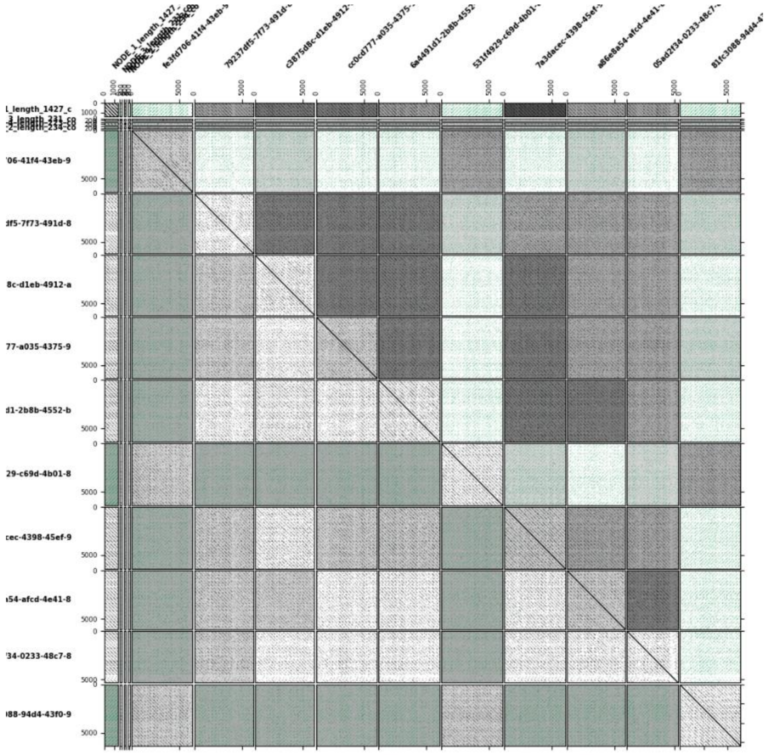

Supercluster

SCL021

Classification

LTR/Ty1-copia/TAR

Assembled NODEs and RE2 contigs

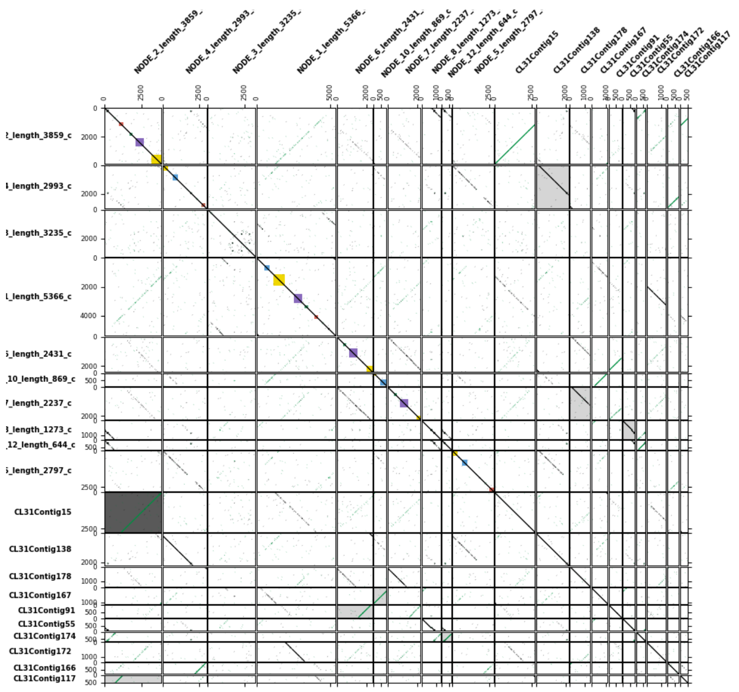

Assembled NODEs and ONT reads (with similar repeat copies)

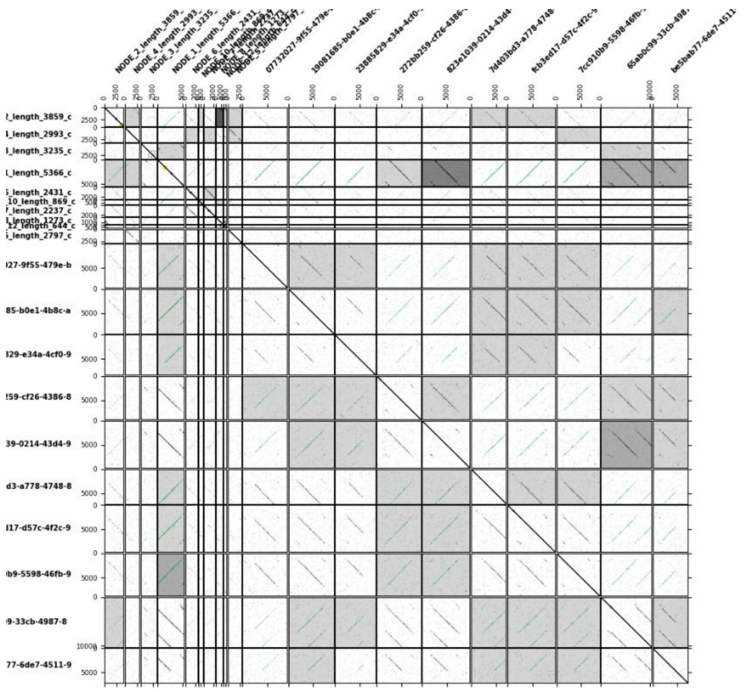

Supercluster | Classification

SCL022 | LTR/Ty1-copia/SIRE

Assembled NODEs and RE2 contigs

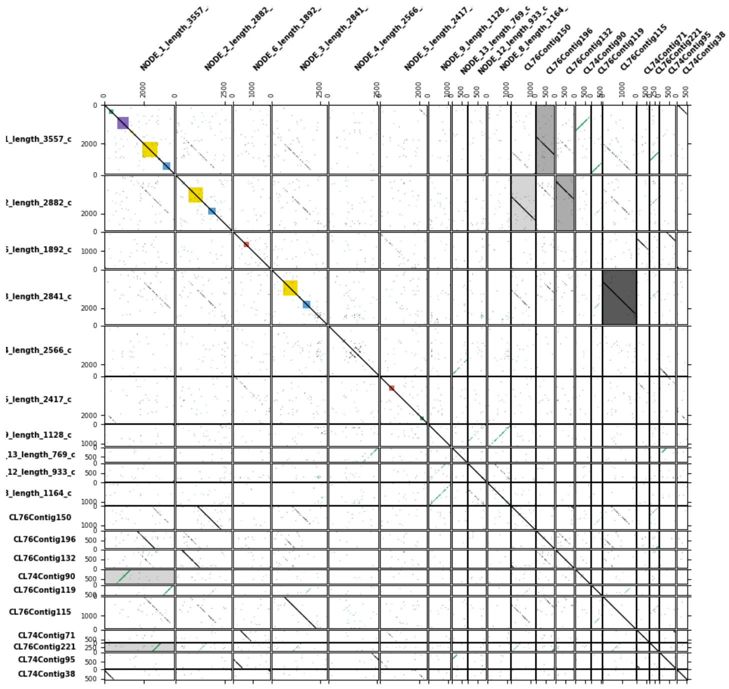


Assembled NODEs and ONT reads (with similar repeat copies)

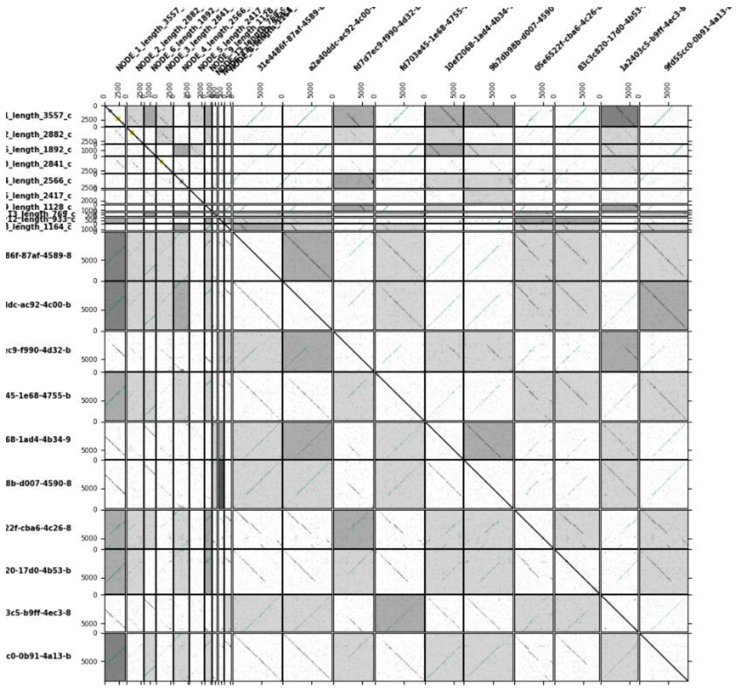

| Supercluster | Classification |
| --- | --- |
| SCL038 | LTR/Ty3-gypsy/CRM |

Assembled NODEs and RE2 contigs

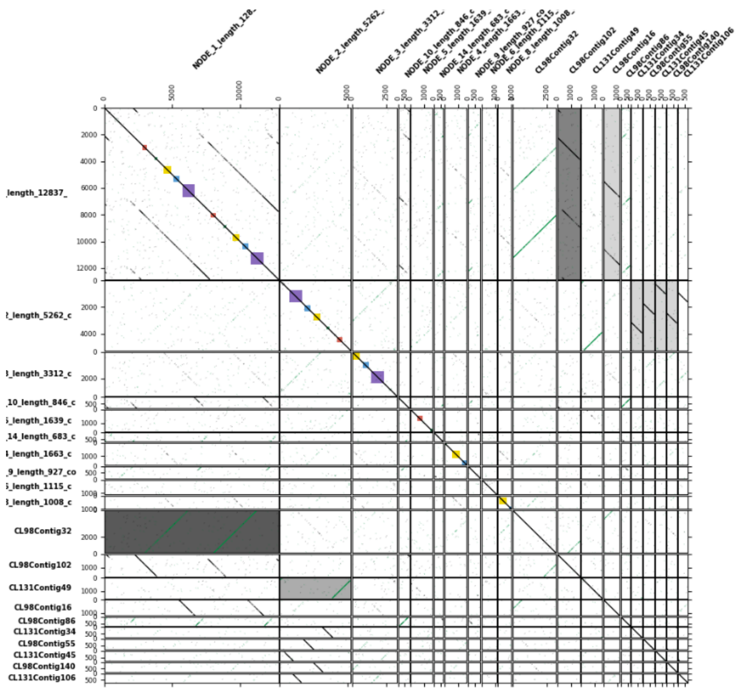


Assembled NODEs and ONT reads (with similar repeat copies)

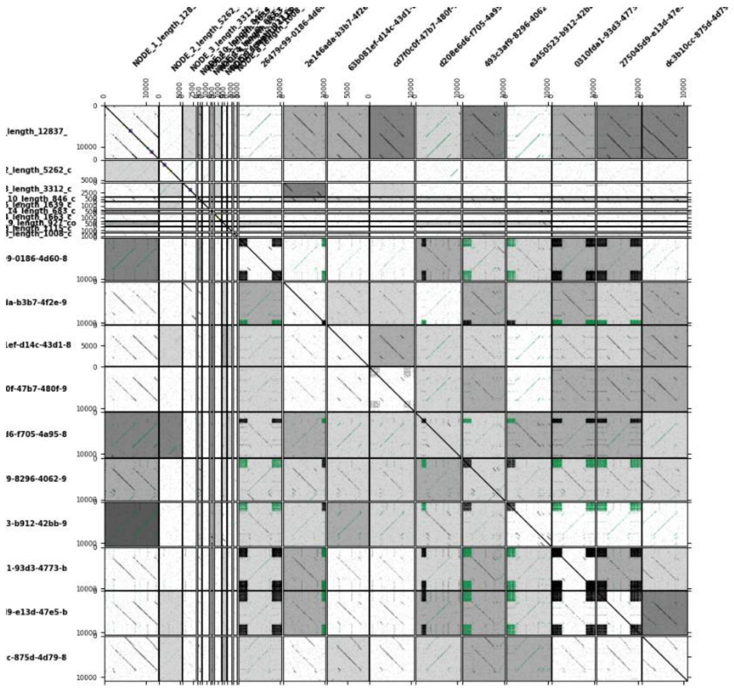

Supercluster | Classification
--- | ---
SCL043 | LTR

Assembled NODEs and RE2 contigs

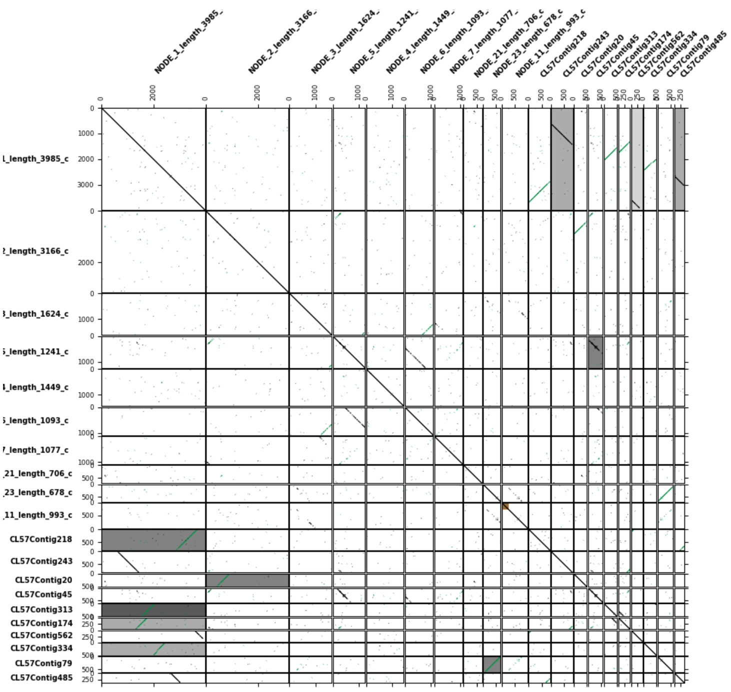


Assembled NODEs and ONT reads (with similar repeat copies)

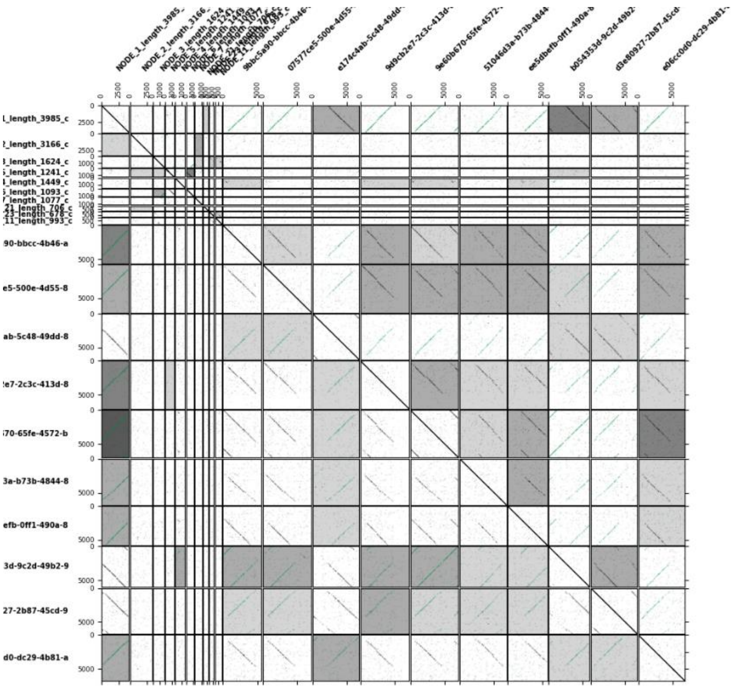

Supercluster Classification

SCL051 pararetrovirus

Assembled NODEs and RE2 contigs

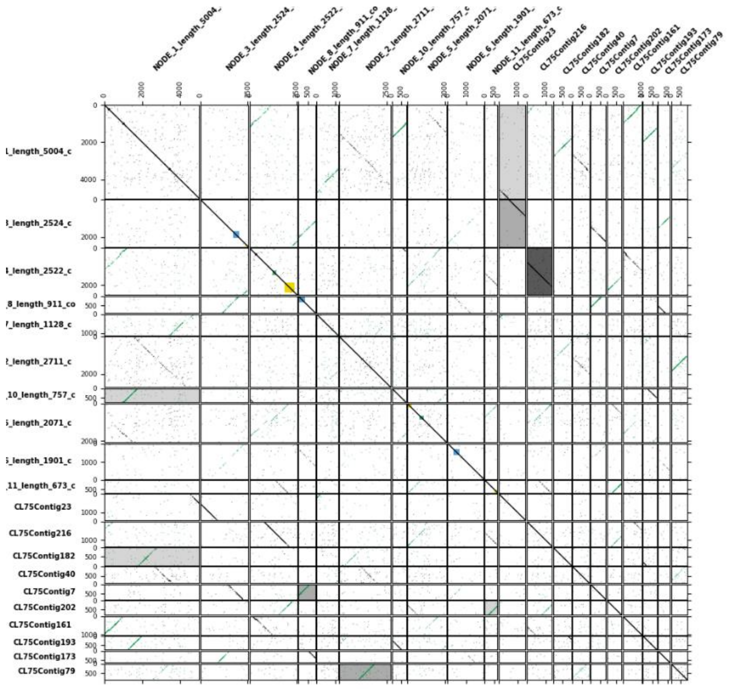

Assembled NODEs and ONT reads (with similar repeat copies)

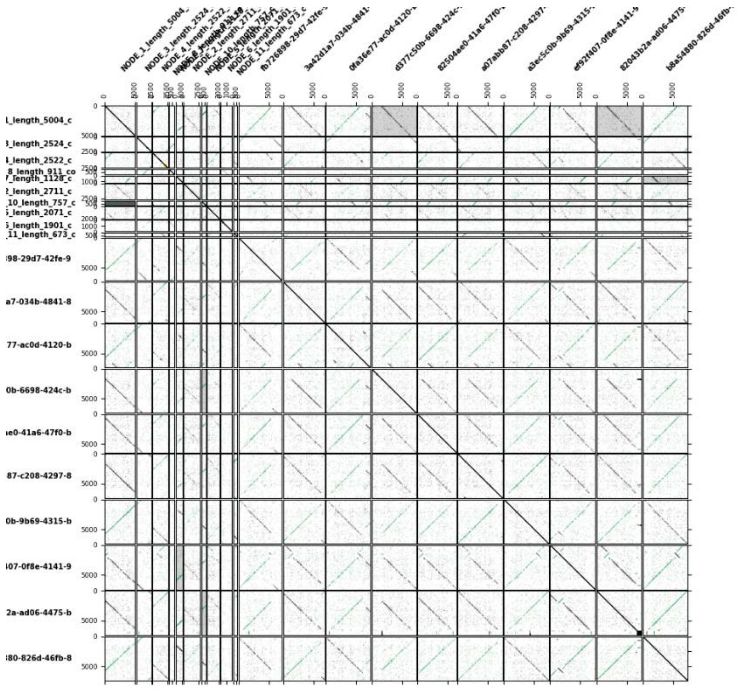

| Supercluster | Classification |
| --- | --- |
| SCL069 | EnSpm_CACTA |

Assembled NODEs and RE2 contigs (Transposase colored in light grey)

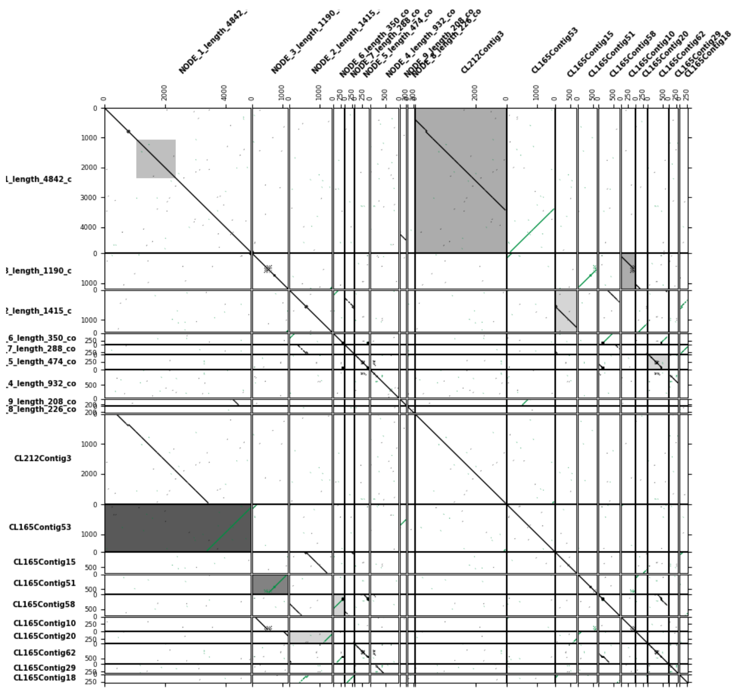


Assembled NODEs and ONT reads (with similar repeat copies)

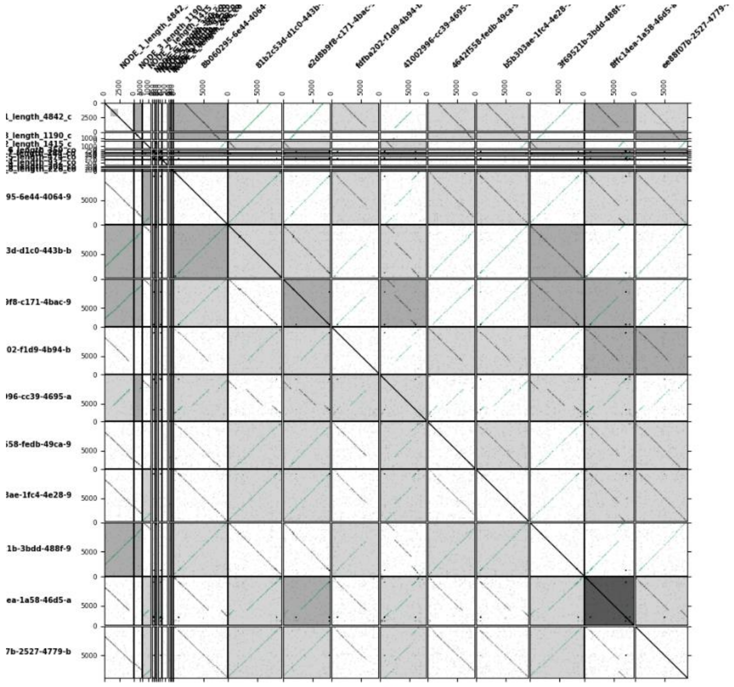

| Supercluster | Classification |
|---|---|
| SCL098 | LTR/Ty3-gypsy/Tekay |

Assembled NODEs and RE2 contigs

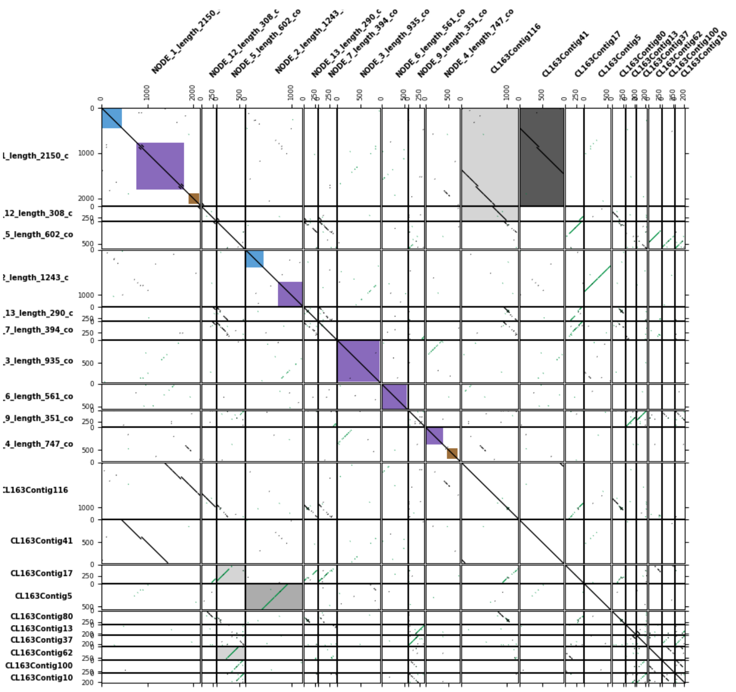

Assembled NODEs and ONT reads (with similar repeat copies)

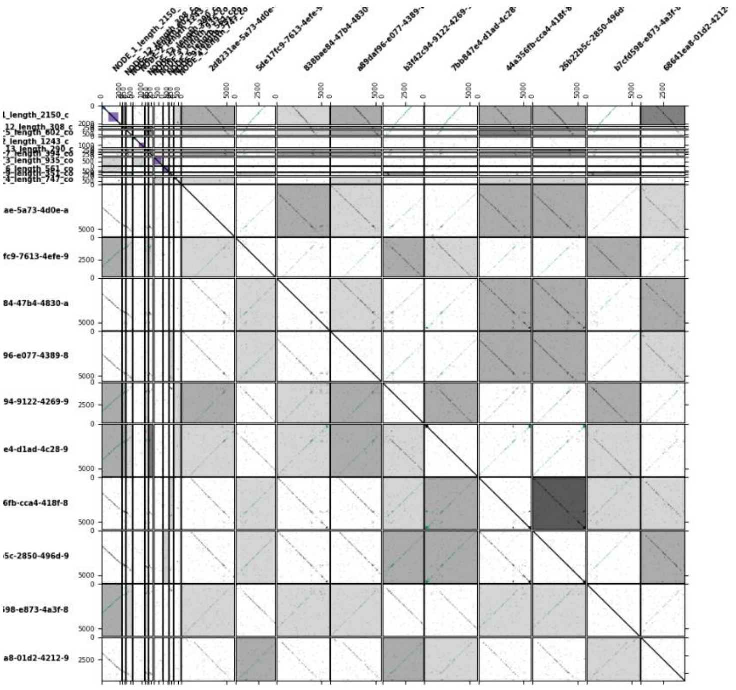

Supplement: Supplementary file 1 — Additional file 1. Collection of dotplots similar to Fig. 2c from various repetitive elements (each represented by a supercluster). [file 12864_2023_9948_MOESM1_ESM.pdf]
